# Univariate versus multivariate spectrophotometric data analysis of triamterene and xipamide; a quantitative and qualitative greenly profiled comparative study

**DOI:** 10.1186/s13065-023-00956-9

**Published:** 2023-05-13

**Authors:** Heidi R. Abd El-Hadi, Maya S. Eissa, Hala E. Zaazaa, Basma M. Eltanany

**Affiliations:** 1 Faculty of Pharmacy, Pharmaceutical Chemistry Department, Egyptian Russian University, Badr City, Cairo Egypt; 2 Faculty of Pharmacy, Analytical Chemistry Department, Cairo University, Kasr El-Aini Street, Cairo, 11562 Egypt

**Keywords:** Antihypertensive drug combination, Green assessment, Multivariate methods, Univariate methods

## Abstract

Triamterene (TRI) and xipamide (XIP) mixture is used as a binary medication of antihypertension which is considered as a major cause of premature death worldwide. The purpose of this research is the quantitative and qualitative analysis of this binary mixture by green univariate and multivariate spectrophotometric methods. Univariate methods were zero order absorption spectra method (D^0^) and Fourier self-deconvolution (FSD), as TRI was directly determined by D^0^ at 367.0 nm in the range (2.00–10.00 µg/mL), where XIP show no interference. While XIP was determined by FSD at 261.0 nm in the range (2.00–8.00 µg/mL), where TRI show zero crossing. Multivariate methods were Partial Least Squares, Principal Component Regression, Artificial Neural Networks, and Multivariate Curve Resolution-Alternating Least Squares. A training set of 25 mixtures with different quantities of the tested components was used to construct and evaluate them, 3 latent variables were displayed using an experimental design. A set of 18 synthetic mixtures with concentrations ranging from (3.00–7.00 µg/mL) for TRI and (2.00–6.00 µg/mL) for XIP, were used to construct the calibration models. A collection of seven synthetic mixtures with various quantities was applied to build the validation models. All the proposed approaches quantitative analyses were evaluated using recoveries as a percentage, root mean square error of prediction, and standard error of prediction. Strong multivariate statistical tools were presented by these models, and they were used to analyze the combined dosage form available on the Egyptian market. The proposed techniques were evaluated in accordance with ICH recommendations, where they are capable of overcoming challenges including spectral overlaps and collinearity. When the suggested approaches and the published one were statistically compared, there was no discernible difference between them. The green analytical method index and eco-scale tools were applied for assessment of the established models greenness. The suggested techniques can be used in product testing laboratories for standard pharmaceutical analysis of the substances being studied.

## Introduction

Applying green reagents in the establishment of ecological analytical techniques has significantly increased in recent years. Green analytical chemistry (GAC) is the practice of minimizing or avoiding the use of potentially harmful chemicals in analytical processes while reducing energy use, waste creation, and other environmental impacts [1, 2]. For quality control professionals, using environmentally friendly techniques for routine drug assessment is of the most important aims and this is what GAC aims to provide. The National Environmental Approaches Index (NEMI), Assessment of Green Profile (AGP), Green Analytical Procedure Index (GAPI), and eco-scale were a few tools for gauging how environmentally friendly a proposed analytical methodology was. These tools were largely based on the 12 guidelines provided by Gauszka et al [3].

**Fig. 1:**
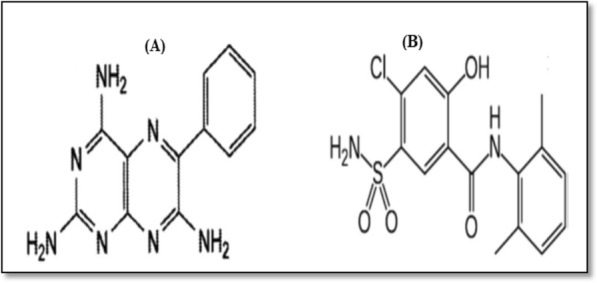
Chemical structure of (**A**) triamterene and (**B**) xipamide

Globally, hypertension affects an estimated 26% of the global populace and the prevalence is expected to rise to 29% by 2025 [4]. Heart attacks and strokes are the most common killers worldwide. They are mostly caused by high blood pressure, which was the most important modifiable risk factor globally in 2013 [5]. There are three medication classes that most commonly used to treat hypertension [6]. The chemical name of triamterene (TRI) is 6-phenylpteridine-2, 4, 7-triamine (Fig. 1) [7]. It is a potassium-sparing diuretic preventing hypokalemia in the body and is also used for the treatment of edema [8]. The chemical name of xipamide (XIP) is 4-chloro-N-(2, 6-dimethyl phenyl)-2-hydroxy-5-sulfamoylbenzamide) (Fig. 1) [7]. It is a diuretic medication used to treat hypertension and shares some characteristics with thiazide diuretics, including its effects on hypokalemia [8]. The combination of TRI and XIP is available in the Egyptian market under the trade name Epitens^®^tablets (each tablet contains 30.0 mg and 10.0 mg for each TRI and XIP, respectively) for treating hypertension.

Reviewing the literature illustrated various spectrophotometric assessments for this mixture in its pharmaceutical formulation for the simultaneous estimation of the combination of TRI and XIP [8–10]. Additionally, various chromatographic techniques for the quantitative evaluation of this mixture were reported [10–12]. A review of the literature also showed that the reported methods did not assess the degree of greenness.

This project aimed to develop first green and accurate univariate and multivariate spectrophotometric approaches for binary mixture analysis of TRI and XIP using UV spectral data in their pure and pharmaceutical formulations and compare between them. Univariate methods such as Zero order (D^0^) and Fourier self-deconvolution (FSD) method, while multivariate approaches, such as, partial least squares (PLS) regression and principal component regression (PCR), artificial neural networks (ANN), and Multivariate Curve Resolution-Alternating Least Squares (MCR-ALS) The current work environmental friendliness helped to reduce any harmful ecological consequences of potentially hazardous materials or study methods. Here, the greenness of the employed chemicals and processes was evaluated utilizing GAPI and penalty points scoring systems. A reliable method, from collection of samples through ultimate analysis, GAPI may provide a comprehensive environmental evaluation of the whole analysis process [13]. Depending on penalty points, the eco-scaling of the developed methodologies was computed and deducted from 100 [14]. ICH techniques were employed to evaluate the established models [15]. The published HPLC approach and the existing methodologies showed no appreciable differences in the statistical analysis.

## Experimental

### Instruments and software

The UV- Visible spectrophotometer JASCO dual beam (Tokyo, Japan) model V-630 was utilized with the included programmer spectra II manager. The spectral slit had a width of 2 nm, and the scanning rate was 1000 nm/min. All chemometric techniques were applied using MATLAB^®^8.3.0.532 (R2014a), PLS Toolbox (version 2.1), MCR-ALS Toolbox, and Neural Network Toolbox (Math Works, United States).

### Materials

Egyptian International Pharmaceutical Industries Co. (EIPICO; Cairo, Egypt) gratefully provided pure samples of TRI and XIP. According to the official method, their purity was found to be 100.13% ±1.03 and 99.25% ± 0.946 for TRI and XIP, respectively [7]. Pharmaceutical dosage form- Epitens^®^tablets were produced by EIPICO. Each tablet contains 30.0 mg and 10.0 mg for each TRI and XIP, respectively, as main components.

### Preparation of standard solutions

Different stock solutions of TRI and XIP (1.00 mg/mL) were created by dissolving 100.0 mg of each drug in 100 mL of methanol. The volume was filled with methanol to make the working solutions for TRI and XIP (100.00 µg/mL) after 10 mL of each drug solution was diluted from its stock standard solutions into two separate 100 mL volumetric flasks. Methanol of analytical grade was purchased from El-Nasr Pharmaceutical Chemicals Co. Cairo, Egypt.

### Procedures

#### Creation of calibration curve

##### Univariate methods

Two isolated sets of 10 ml volumetric flasks were used to accurately transfer multiple aliquots of TRI and XIP from each stock standard solution in order to create linearity ranges (2.00–10.00 µg/mL) and (2.00–8.00 µg/mL) for TRI and XIP, respectively.

- Zero order absorption spectra method

The TRI and XIP absorption spectra were acquired spanning the wavelength range of 200.0 to 400.0 nm. The linear regression was calculated after the calibration curve was drawn between both the absorbance at 367.0 nm and the appropriate TRI concentrations.

- Fourier self-deconvolution spectrophotometric method

Utilizing the FSD function, each drug spectra was analyzed and deconvoluted separately. The amplitude of the substance deconvoluted signal at 261.0 nm was plotted to produce the calibration curve for XIP in binary mixture. The pharmaceutical formulation form and laboratory-prepared mixtures were both generally calculated using the linear relationship that was generated.

##### Multivariate methods

Both calibration and validation datasets were created utilizing the five-level, two-factor experimental design and five concentration levels labelled from + 2 to -2 for each of the two medications under evaluation. Concentration ranges of TRI and XIP which used for calibration and validation sets were (3.00–7.00 µg/mL) and (2.00–6.00 µg/mL) respectively. 25 samples comprising mixtures of TRI and XIP in varied ratios were created by transferring various concentrations of TRI and XIP from their corresponding working solutions to a series of 10-mL volumetric flasks. To assess the predictive power of each built calibration model, seven samples were randomly chosen for the cross validation set and the rest of them were utilized to build the regression model. With 0.1 nm interval, the absorption spectra of the calibration and validation sets were scanned at wavelength range of 220.0–240.0 nm. The results were then imported into MATLAB^®^to be used in multivariate calibration models and additional data processing, including PLS, PCR, ANN, and MCR-ALS, were built. Each technique parameter was examined and improved. The two medications were then determined jointly using the cross validation set and the previously defined criteria for each model.

- Principal component regression and partial least squares

With the aid of mean-centered data and leave-one-out cross validation, the number of latent variables (LVs) and principal component (PC) in the PLS and PCR, respectively calibration models were altered. The ideal number of LVs and PC to achieve the lowest root mean square calibration error (RMSEC) was three.

- Artificial neural networks

A feed-forward model was trained to enhance the ANN calibration method. There were 201 and 2 neurons in the input and output layers, respectively. It was also attempted to optimize the hidden layer’s number of neurons. Purelin-purelin transfer function, which is often employed for linear processes, was utilized to choose 8 neurons from the hidden layer. Furthermore, 100 epochs were chosen as the optimal number.

- Multivariate curve resolution-alternating least squares

The most crucial optimization factor in MCR-ALS calibration was the imposed constraints; non-negativity constraints, which were obtained using the fast non-negativity constrained least squares algorithm (fnnl), were implemented to both concentration and spectral profiles in order to obtain optimum features with the fewest iterations.

#### Laboratory prepared mixtures analysis

The ratios of five different laboratory TRI and XIP combinations were created in two various sets, and the measurements were taken between 200.0 and 400.0 nm for each set. To quantify the concentration of each medication individually, the aforementioned techniques were applied. The steps outlined below (building calibration curves for univariate methods) were adhered to, and the associated linear regression was employed to estimate each drug’s concentration.

#### Analysis of a drug formulation

A clean mortar and pestle was utilized to weigh, properly crush, and mix 20 Epitens^®^pills. The exact amount of smashed powder needed to make a solution with 1000.0 µg/mL was put into a 25 mL volumetric flask, and then 15 mL of methanol was added. The created solution was cooled, diluted to volume with methanol, and filtered after being sonicated for 30 min. To achieve the right dilutions and final concentrations of 6.00 µg/mL for TRI and 2.00 µg/mL for XIP, respectively, the correct dilutions were made using the same solvent. Following the aforementioned methods, each drug’s concentration was calculated using the associated regression equations. In their tablet, the developed methodologies were successful in recognizing TRI and XIP.

## Results and discussion

Comparing spectrophotometric approaches to others that require complex equipment or chemical pretreatment, such as chromatographic techniques, spectrophotometric methods have the benefits of being quick, easy, and inexpensive [16]. The current study offers a simple, environmentally friendly UV spectrophotometric techniques for assessing TRI and XIP simultaneously in pharmaceutical and bulk forms.

**Fig. 2:**
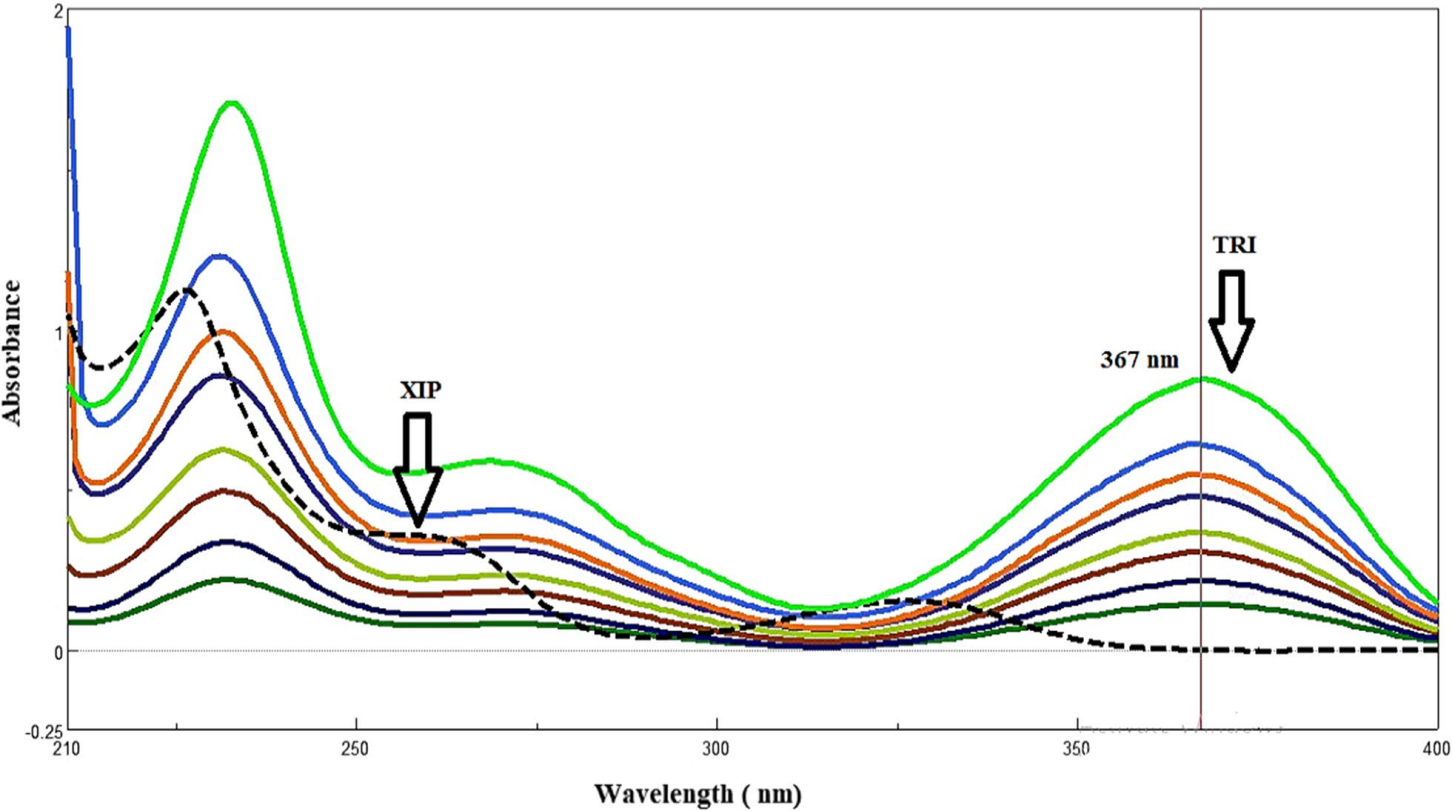
Zero order over extended absorption spectra of triamterene (2.00–10.00 µg/ml) at 367.0 nm in presence of xipamide (8.00 µg/ml) zero-order spectra using methanol as a solvent

A quantitative measurement of TRI was made at zero-order spectra at 367.0 nm (Fig. 2), and XIP was resolved and quantified by FSD method at 261.0 nm. The FSD approach tends to reduce excessively overlapping spectra, enhancing the resolution of overlapping spectra by eliminating the broadening impact caused by the spectrophotometer convolution [17].

Multivariate approaches can be used to quickly predict analytes concentrations by evaluating spectra from unknown chemicals using a multivariate model [18]. Numerous spectrophotometric techniques have been previously published for evaluating TRI and XIP in dosage forms, but there is no reported chemometric methods till nowadays. Here, the proposed techniques’ “greenness” was evaluated using a brand-new and powerful scoring method called the “greenness assessment tool.“

### Univariate method

#### Zero order absorption spectra method

It is the simplest way for evaluating a mixture since one component can be detected directly and the other medicines do not interact with it [19, 20]. Spectra in (Fig. 2) showed that TRI was extended than XIP so it can be measured in zero absorption spectra at 367.0 nm. While XIP showed no interference, TRI was assessed over the linearity range (2.00–10.00 µg/mL) and detected at 367.0 nm.

#### Fourier self-deconvolution spectrophotometric method

**Fig. 3:**
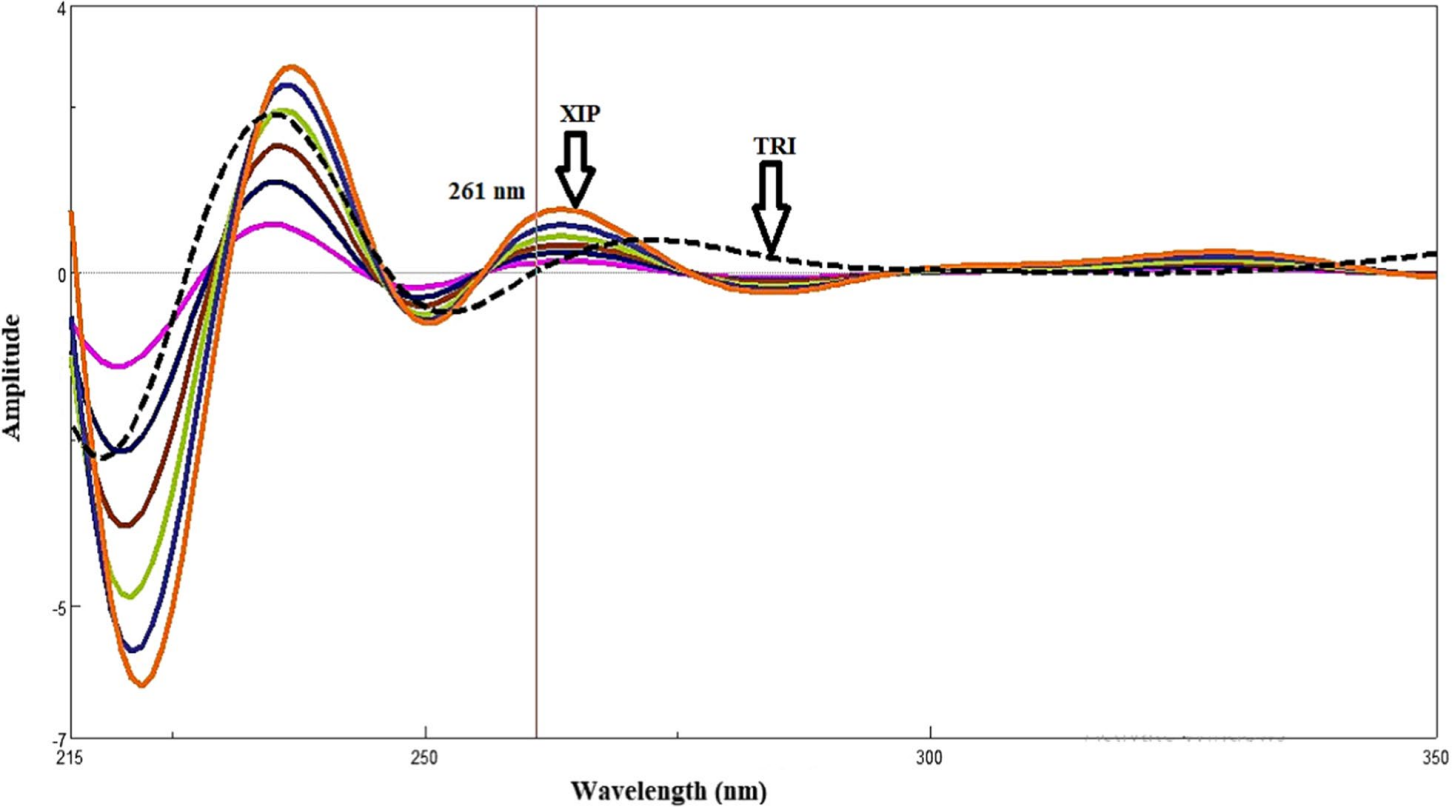
Deconvoluted spectra of xipamide (2.00–8.00 µg/ml) determined at 261.0 nm, zero-crossing point of triamterene deconvoluted spectrum using methanol as a solvent

FSD is recognized as an efficient mathematical computing technique that is applied in a number of signal processing applications. The physical convolution brought on by instrumental distortions to spectrum signals was eliminated by the currently employed technique. What gives the current methodology its resolving capacity is the evolution of major spectral zones with zero-crossing or no contribution points between the spectra of mixed components [17]. Reversing the physical convoluting, distorting impacts from the spectrophotometer performed to the recorded spectrum is the underlying idea behind FSD [21]. The resolution and determination of TRI and XIP in their binary mixture were described by the current FSD method. Since TRI overextended than XIP, as TRI zero-order spectrum was previously calculated. The spectra manager software’s full width at half maximum value (FWHM) function was used to automatically deconvolute the zero-order spectra of XIP and TRI with FWHM value of 65. XIP calibration curve was plotted at 261.00 nm over linearity range (2.00–8.00 µg/mL) was the respective zero-crossing point for TRI as shown in (Fig. 3). By examining the laboratory-prepared mixtures with various TRI and XIP ratios, the specificity of the suggested methods was evaluated. The obtained results were satisfactory were as shown in (Table 1).

**Table 1:** Determination of TRI and XIP in their laboratory prepared mixtures by the proposed univariate methods

Claimed concentration taken (µg/ mL)		TRI	XIP
TRI	XIP	D^0^ Recovery (%)	FSD Recovery (%)
**5.00**	**4.00**	100.87	99.95
**6.00**	2.00*	99.09	99.85
**4.00**	**3.00**	98.90	100.64
**6.00**	**6.00**	100.88	101.84
**3.00**	**5.00**	101.90	100.86
**Mean (%)**		100.33	100.63
**SD**		1.28	0.80

Bold values represent concentrations of developed drugs (TRI and XIP) in prepared lab mixtures while mean and SD are represent average and standard deviation of recoveries % values

*The same ratio in dosage form

### Multivariate methods

The main advantage of multivariate analysis is that it considers more than one factor in data analysis. It looks at the various independent variables that influence the dependent variable, not like univariate analysis which depend on one variable [18, 22]. Using multivariate data processing, the substantially overlapping drug spectra were separated. The investigation of TRI and XIP used four multivariate techniques. For the multivariate calibration to produce the best predictions, a detailed experimental design of the calibration set’s composition is required. To construct the samples, a multilevel multifactor design was utilized, with 18 samples serving as a training set and the remaining 7 samples serving as a validation set (Table 2). The range of spectra between 220.0 and 240.0 nm, each scanned at 0.1 nm, produced the best results. 201 experimental points were utilized to calibrate and evaluate the established models. To predict unknown samples, calibration models were proposed, tested, and used.

**Table 2:** Concentrations TRI and XIP in the calibration and validation sets for the multivariate calibrations

Mix no	Concentration (µg/mL)	
	TRI	XIP
1	5.00	4.00
2	5.00	2.00
3	3.00	2.00
4	3.00	6.00
5	7.00	3.00
6	4.00	6.00
7	7.00	4.00
8	5.00	3.00
9	4.00	3.00
10	4.00	5.00
11	6.00	6.00
12	7.00	5.00
13	6.00	4.00
14	5.00	6.00
15	7.00	6.00
16	7.00	2.00
17	3.00	5.00
18	6.00	2.00
19	3.00	4.00
20	5.00	5.00
21	6.00	5.00
22	6.00	3.00
23	4.00	2.00
24	3.00	3.00
25	4.00	4.00

The highlighted rows represent the validation set

#### Principal component regression and partial least squares

**Fig. 4:**
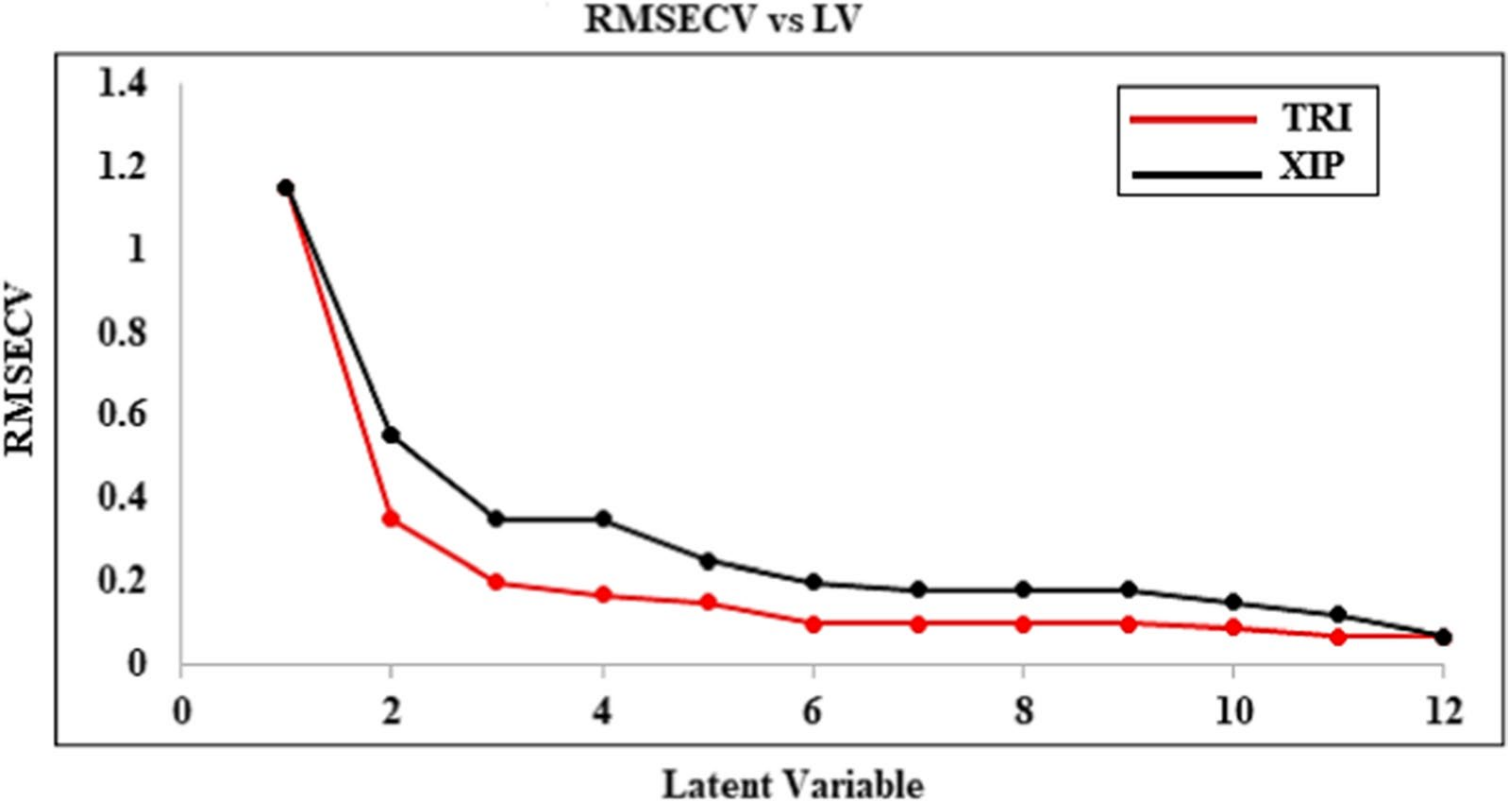
RMSEC plot of the cross validation results of the calibration set as a function of the number of latent variables used to construct partial least squares calibration

**Fig. 5:**
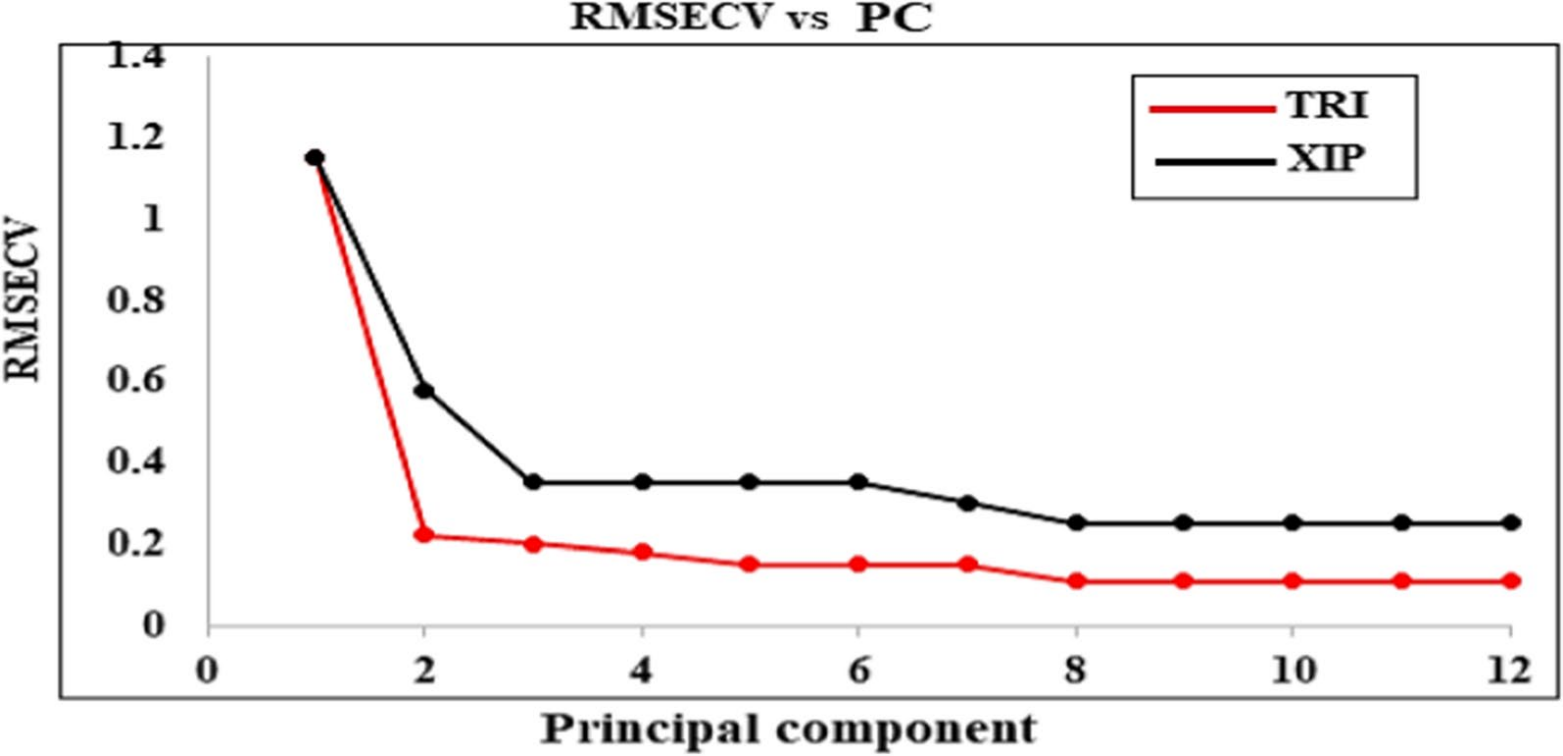
RMSEC plot of the cross validation results of the calibration set as a function of the principal component used to construct principal component regression calibration

The most frequently employed inverse least squares for building multivariate calibration models are PCR and PLS [23–25]. In the statistical study of spectra, PLS and PCR algorithms are frequently employed to extract detailed data from more general data [26, 27]. The PLS and PCR algorithms simultaneously consider the data about replies and levels [28, 29]. The leave one out cross-validation method was used in this research to determine the appropriate number of components by excluding one sample at a time. The optimal number of LVs is the one that results in the fewest significant predictions errors [25, 30]. In this case, three latent variables were shown to be the optimum number. (Figs. 4 and 5).

#### Artificial neural networks

**Fig. 6:**
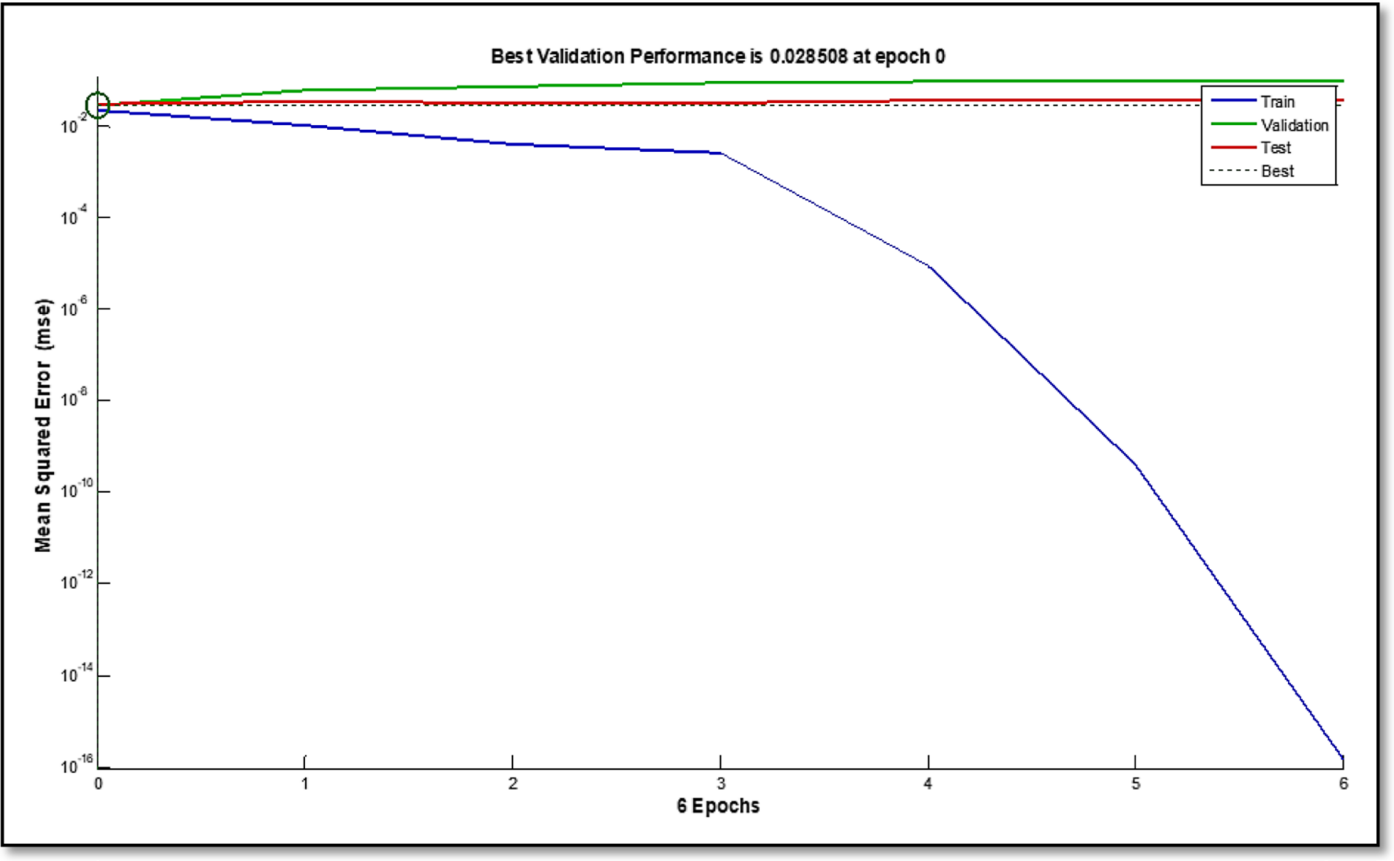
Best validation performance for the prediction of artificial neural networks model

**Fig. 7:**
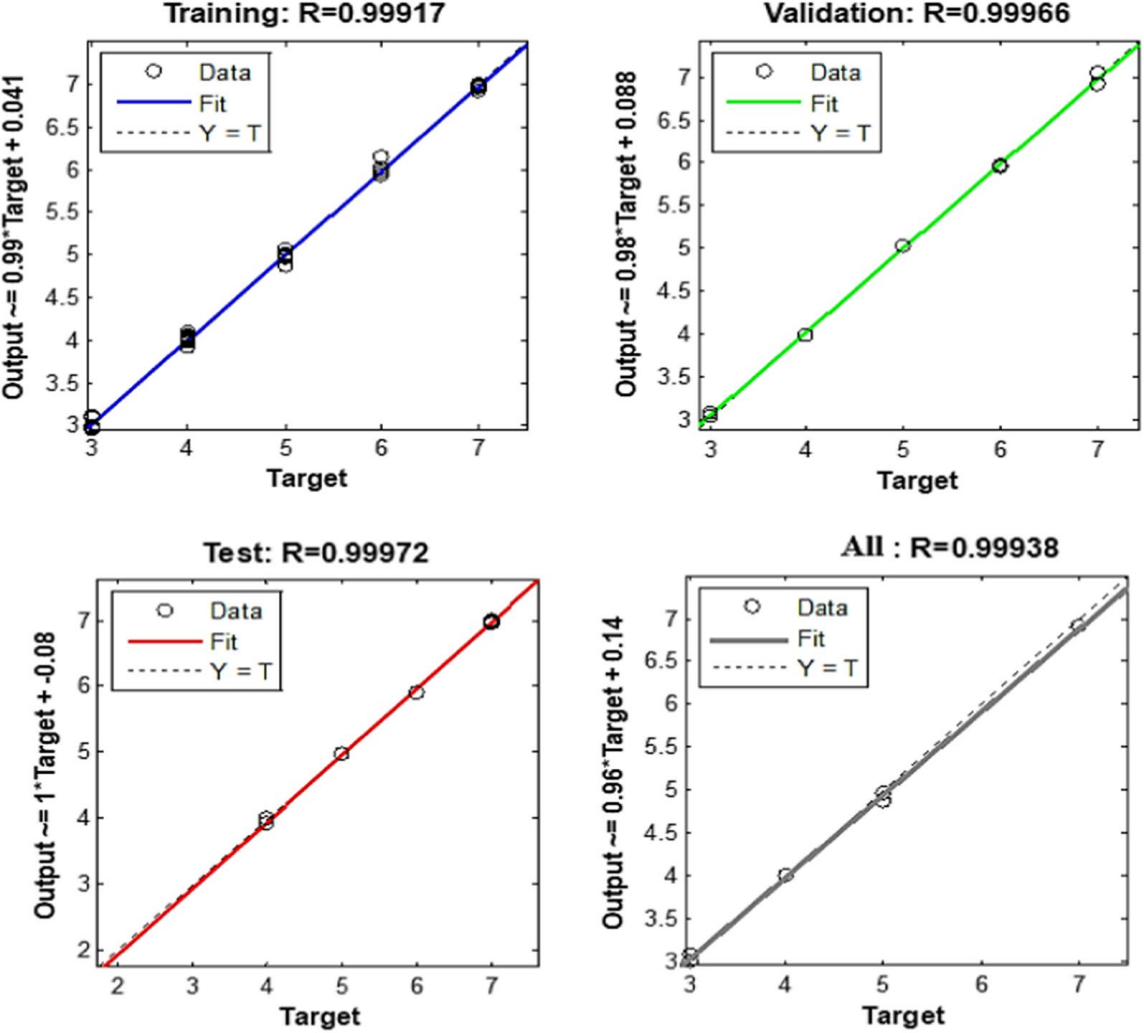
Prediction for the training, test and validation diagrams of artificial neural networks model

ANN is a method for imitating intelligence that finds links between inputs and outputs by using a large number of simple, densely linked nodes or artificial neurons. Three layers input, hidden, and transfer functions make up an ANN typically [31, 32]. The feed-forward model that was trained is the ANN type that was employed in this study. Two neurons were utilized in the output layer, equivalent to the number of components intended to be evaluated in each sample, and 201 neurons were utilized as the input layer, representing how many spectral data points were applied. Trial-and-error should be employed to alter the hidden layer’s number of neurons. With 100 epochs and a purelin-purelin transfer function, 8 hidden neurons were the ideal number. The output plot of an appropriately trained ANN for mean squared error (MSE) versus epochs is shown in (Fig. 6). The MSE of training gradually dropped after epoch = 0. The trial and validating curves were similar and showed no abrupt changes, supporting the idea that there was no overfitting. In (Fig. 7), prediction diagrams for the training, test, and validation series of the chosen layers and neurons are also shown. For validation, test and training series, the high effectiveness of this model in prediction is indicated by a correlation value (R) that is near to 1 [33].

#### Multivariate curve resolution-alternating least squares

Factor analysis is utilized to develop the multi-wavelength extension of Beer’s-Lambert law, and MCR implies a bilinear model [31]. The spectral and concentration -profile matrices of each drug in the samples are separated out from the measured spectra data matrix in MCR, and error is then determined [32, 34]. The ALS approach was optimized for repeated concentration predications from spectral profiles and vice versa. Data matrix decomposition does not have a unique solution, hence by placing limitations like uni-modality, closure, equality, or non-negativity, it is possible to decrease the number of alternative solutions.

“simple-to-use interactive self-modeling analysis” technique was used to estimate spectral-profile matrix to begin the optimization of ALS [35]. Additionally, the unconstrained least-squares solution for the concentration profile was computed using the spectral-profile matrix.

**Fig. 8:**
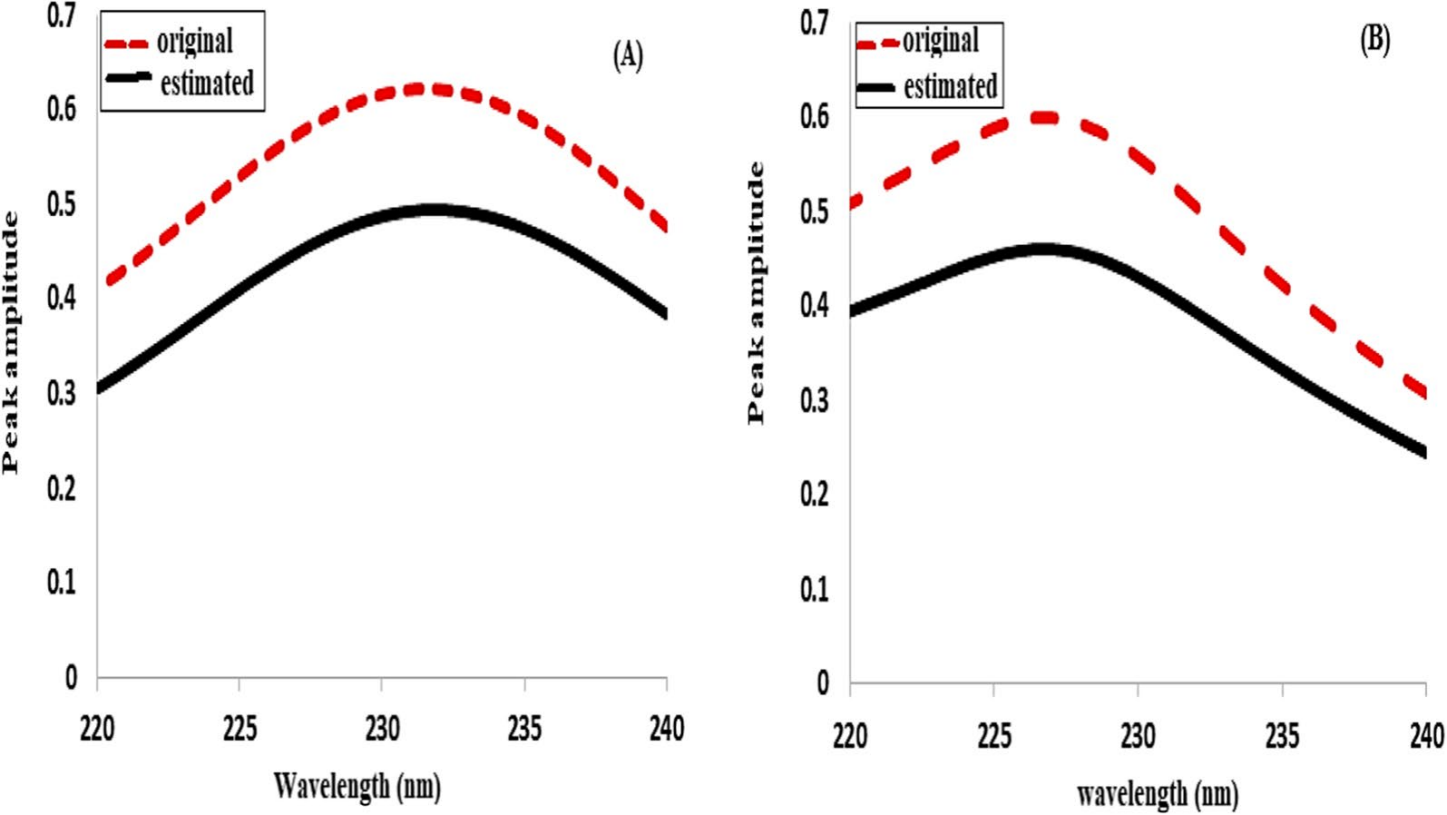
Original spectra and estimated spectra by multivariate curve resolution-alternating least squares of (**A**) triamterene and (**B**) xipamide

In the present work, both concentration and spectral profiles were subjected to non-negativity constraints. Both concentration and spectra have to equal 2 to satisfy the non-negativity constraints. When a specific convergence threshold was reached, the ALS optimization process came to an end (30%). The convergence is frequently stopped (typically set at 0.1%) when the variation between the root mean square of “E” the residuals matrix, between multiple iterations, is lower than a threshold level [33]. Iterations processed till an ideal result is found which satisfies both the set convergence criteria and the hypothesized limitations. At two iterations, the convergence was interrupted. The computed percentages of variance (R^2^) and lack of fit (% lof) were 99.9829 and 1.3084, respectively, and they were sufficient to support the effectiveness of the provided MCR-ALS model. These model could be used to estimate the spectral profile of medications. The calculated spectra are seen to resemble the original ones (Fig. 8). The values of the recoveries, mean recoveries, and RSD % are shown in (Table 3).

**Table 3:** Prediction of validation set samples using the proposed multivariate methods

Concentration (µg/mL)		PLS		PCR		ANN		MCR-ALS	
TRI	XIP	TRI	XIP	TRI	XIP	TRI	XIP	TRI	XIP
4.00	6.00	101.55	98.22	101.56	98.22	99.75	100.16	98.75	96.66
6.00	6.00	100.02	97.22	100.03	97.21	98.80	98.95	97.52	97.26
7.00	5.00	97.58	102.29	97.58	102.29	99.71	99.70	98.59	98.06
6.00	4.00	101.23	99.96	101.23	99.96	101.30	100.10	100.79	97.75
5.00	6.00	100.71	97.72	100.71	98.71	99.38	100.03	98.92	98.39
7.00	2.00	100.81	100.17	100.81	100.17	99.49	100.19	102.11	99.21
6.00	2.00	103.53	101.22	102.23	101.23	100.90	101.54	101.21	99.16
Mean (%)		100.78	99.54	100.59	99.68	99.90	100.10	99.70	98.07
RSD (%)		1.77	1.89	1.48	1.76	0.81	0.71	1.67	0.96

Table 4 provides the regression and validation parameters for the proposed spectrophotometric methods for determining pure TRI and XIP samples, the validation process followed ICH criteria [15]. Sensitivity is the change in the response of a measuring instrument divided by corresponding change in the stimulus or simply the gradient of the calibration function; the lower the LOD and LOQ, the higher the sensitivity [15]. Values of LOD and LOQ for developed methods were mentioned in (Table 4) while values of LOD for literature method were 0.58 and 0.67 for TRI and XIP, respectively and LOQ values were 1.78 and 2.03 for TRI and XIP, respectively. Root mean square error of prediction (RMSEP) and RMSEC and for each component were also computed by the proposed multivariate models.

**Table 4:** Regression and validation parameters of the established univariate and multivariate methods for determination of TRI and XIP

Validation parameters	TRI					XIP				
	D^0^	PLS	PCR	ANN	MCR-ALS	FSD	PLS	PCR	ANN	MCR-ALS
Linearity range (µg/mL)	2.00–10.00	3.00–7.00				2.00–8.00	2.00–6.00			
Correlation coefficient (r)	0.9996	0.9998	0.9995	0.9998	0.9998	0.9992	0.9997	0.9998	0.9996	0.9997
Slope	0.0843	1.015	1.0116	1.0335	0.9996	0.1083	1.0197	1.0457	0.99658	1.0375
Intercept	− 0.035	− 0.181	− 0.169	− 0.3155	− 0.147	-0.092	− 0.1716	− 0.3336	− 0.11378	− 0.3393
RMSEC	–	0.162	0.053	0.156	0.150	–	0.146	0.103	0.136	0.099
RMSEP	–	0.138	0.017	0.104	0.011	–	0.085	0.094	0.144	0.112
Accuracy ^a^ (Mean% ± SD)	99.49 ± 1.37	–	–	–	–	100.05 ± 1.28	–	-	-	-
Precision^b^										
Repeatability	1.01	–	–	–	–	1.12	–	–	–	–
Intermediate	0.77	–	–	–	–	1.62	–	–	–	–
LOD^c^	0.22	0.19	0.28	0.14	0.15	0.31	0.20	0.16	0.22	0.16
LOQ^c^	0.66	0.57	0.84	0.42	0.45	0.93	0.60	0.48	0.66	0.48

a Average of three different concentrations repeated three times within the day

b Precision was evaluated by measuring the response of three concentrations of each drug three separate times on the same day (repeatability) and on three different days (intermediate precision)

c LOD and LOQ were calculated from the standard deviation (s) of the response and the slope of the calibration curve (S) according to the following equations: LOD = 3.3(s/S) and

LOQ= 10(s/S)

### Greenness assessment

In order to assess whether different analytical techniques adhered to the green chemistry theory, it was crucial to evaluate their environmental impact. Various techniques can be employed to evaluate the greenness of everything [36]. Ecological impact of analytical techniques was assessed using four primary factors: high energy consumption, high waste production, excessive chemical use and its dangers, and the use of large amounts of chemicals [37, 38]. The greenness of the suggested technique was assessed in addition to its cost-effectiveness utilizing two analytical tools, and they were as follows:

### Green analytical procedure index

**Fig. 9:**
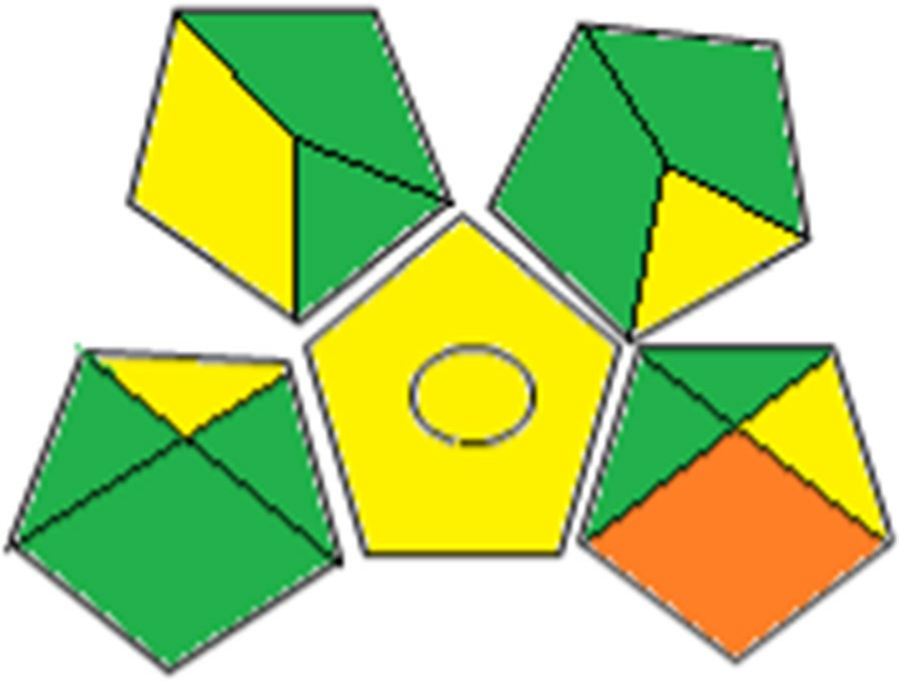
GAPI pictograms for six proposed methods

Wasylka [3] proposed a noval metric for evaluating greenness. It is a tool that provides a wealth of knowledge on fifteen areas of analytical techniques, each of which is symbolized by five pentagrams. Red indicates a significant environmental risk, yellow indicates to lesser ecological tolerance while green indicates higher ecological tolerance according to the GAPI color scheme [3, 39]. Analytical procedures examined by the GAPI tool were given a green evaluation profile as shown in (Fig. 9). It shows that the established methods were a simple, environmentally friendly methods that could be used for both characterization and measurement without the requirement for extraction strategies. Additionally, it offered uncomplicated processes that generated the least quantity of waste and dangerous substances.

### Analytical eco-scale

It is feasible to compare and choose the greenest option by using an analytical technique called the eco-scale [40, 41]. It uses a basis of 100 penalty points. Penalty points are determined for each analytical technique parameter and reduced from 100. The higher the score, the more ecofriendly and economically efficient the analytical technique [14]. (Table 5) demonstrated that the proposed methodologies provide 11 penalty points, which indicates excellent green analysis with fewer waste and potentially harmful reagents.

**Table 5:** Penalty points (PPs) for six proposed methods

Parameters	Established methods
**Reagents**	
Methanol	6
**Instrument**	
Energy (˃ 0.1kWh per sample)	0
Occupational hazard	0
Waste	1
**Total PPs**	Ʃ7
	**93**
**Analytical Eco-scale score**	**Excellent green analysis**

Bold values represent the main factors (reagent and instrument) that affect greenness assesment of methods and to their scores which represented by pps

Analytical eco−scale score = 100 (the ideal score of green analytical method)

Analytical eco−scale score> 75 (excellent green analysis)

Analytical eco−scale score 50–75 (the green analysis is acceptable)

Analytical eco−scale score< 50 (the green analysis is inadequate)

### Comparative statistical study

For the accurate estimation of TRI and XIP in Epitens^®^ tablets D^0^, FSD, PLS, PCR, ANN, and MCR-ALS techniques were applied. Statistics were used to evaluate the result of the established regression techniques to those produced using the reported technique [11]. The recommended methodologies and the reference method showed great agreement, as shown by the F-ratio test and t-test. The differences between the two experiments’ results were found to be insignificant, and all findings were collected in (Table 6).

**Table 6:** Statistical analysis of the results obtained by the developed univariate, multivariate methods and the reported HPLC methods for the determination of TRI and XIP in pharmaceutical preparation

Parameters	TRI						XIP					
	Proposed methods					Reported method	Proposed methods					Reported method
	D^0^	PLS	PCR	ANN	MCR-ALS	HPLC ^a^	FSD	PLS	PCR	ANN	MCR-ALS	HPLC^a^
Mean ^b^ (%)	100.29	99.06	100.17	101.25	100.25	102.00	99.88	100.30	99.12	98.12	100.54	101.00
SD	0.46	0.58	0.46	0.60	0.52	0.33	0.75	0.81	0.72	0.92	0.89	0.62
Variance	0.21	0.33	0.21	0.36	0.27	0.11	0.56	0.65	0.52	0.85	0.80	0.38
n	6	6	6	6	6	6	6	6	6	6	6	6
Student’s *t*-teste (2.23)^c^	0.14	0.09	0.13	0.37	0.15	–	0.36	0.60	0.21	0.16	0.98	–
*F*-value (5.05)^c^	1.89	2.99	1.88	3.19	2.47	–	1.46	1.69	1.35	2.20	2.07	–

aHPLC method: phosphate buffer pH 3.5 and acetonitrile (30:70% v/v) at flow rate of 1.0 mL/min, detection at 254.0 nm (11)

bAverage of 6 experiments

c Figures between parentheses represent the corresponding tabulated values of t and F at P=0.05

When the described techniques were applied to the preparation of pharmaceuticals, the recovery % data were compared statistically using one way-ANOVA; no substantial differences were found (Table 7). These results support the applicability of the developed models for the precise estimation of TRI and XIP in their pharmaceutical preparation.

**Table 7:** One-way ANOVA statistical analysis within 95% confidence interval on the recovery percentage data obtained from the application of the proposed methods and the reported HPLC method on Epitens^®^ tablets tablet

Source of variation	SS	df	MS	f	P-value	F-crit
TRI						
Between groups	9.77	5	1.95	2.13	0.08	2.53
Within groups	27.45	30	0.91	-	–	–
XIP						
Between groups	3.37	5	0.67	1.95	0.11	2.53
Within groups	10.36	30	0.34	–	–	–

**Fig. 10:**
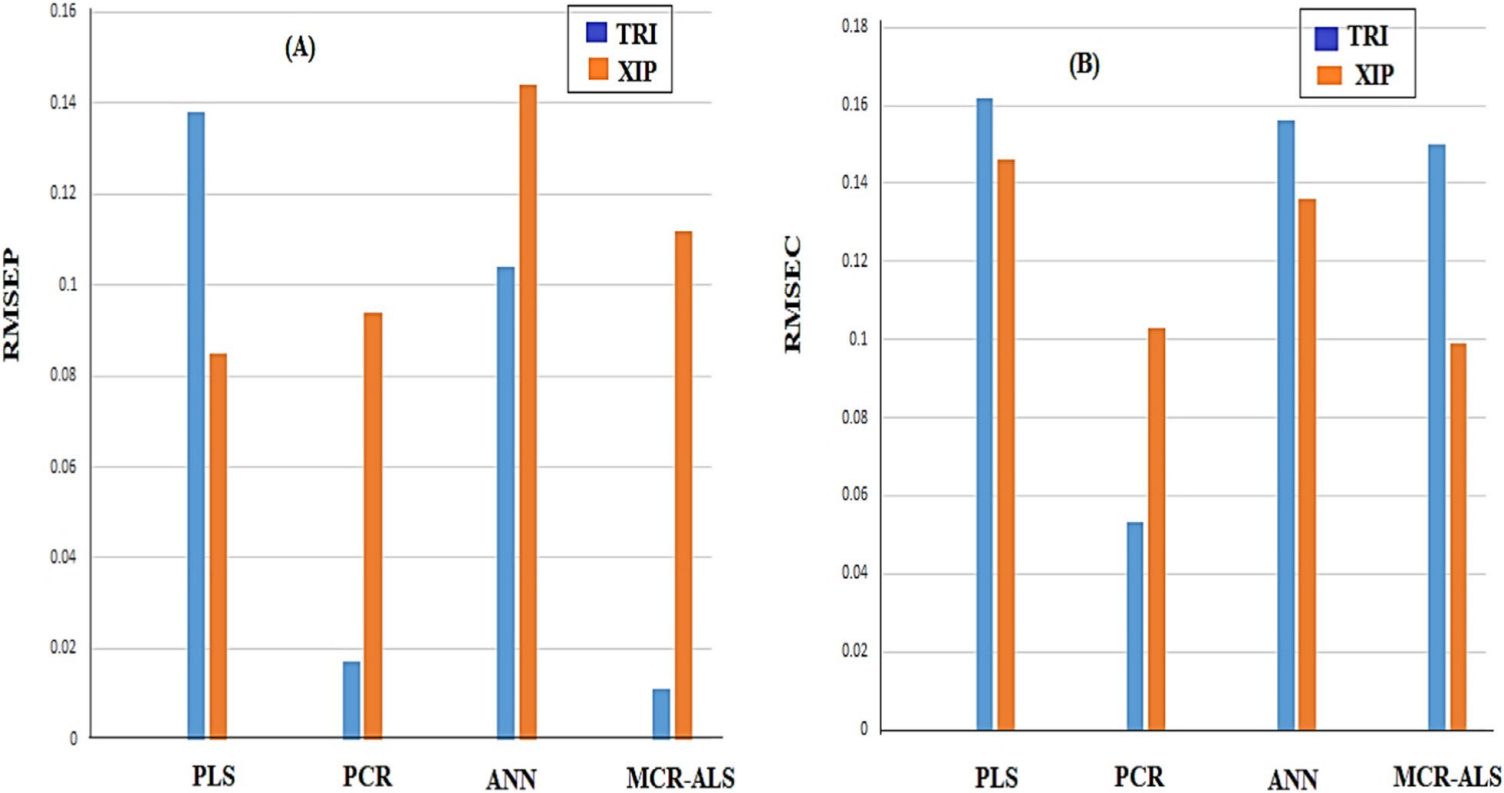
The calculated (**A**) root mean square error of prediction for each component obtained by the proposed validation models and (**B**) root mean square error of calibration obtained by the corresponding calibration model

Column charts show the RMSEP and RMSEC calculated using the suggested validation and calibration models for each ingredient (Fig. 10). The PCR model has the lowest RMSEP and RMSEC, per the findings. To demonstrating the method’s precision and reliability, RMSEP is a diagnostic tool for evaluating prediction mistakes. RMSEP measures the range of concentration mistakes and acts as a standard deviation [42]. The optimal model for determining a numerical ingredient was found to be the PCR model.

## Conclusion

The suggested spectrophotometric techniques can be utilized for the concurrent evaluation of the studied binary mixture in dosage form using straightforward procedures and minimal manipulation. The two compound in their standard and medicinal forms could be analyzed using the suggested green methods quickly, easily, and with excellent sensitivity and reliability. The current methodologies greenness was taken into consideration during the early stages of their development, and it was subsequently evaluated utilizing the GAPI index and the penalty point scoring system. statistically, the proposed and reported methods did not significantly differ, demonstrating the sensitivity, accuracy, and precision of both. The six recommended strategies, however, are less troublesome and more appropriate for determining the drug mixture under study.

## Data Availability

Spectrophotometric data obtained from spectrophotometer software. Datasets generated and/or analyzed during the current study are available from the corresponding author on reasonable request.

## Abbreviations

TRI: Triamterene
XIP: Xipamide
D^0^: Zero order absorption spectra method
FSD: Fourier self-deconvolution
GAC: Green analytical chemistry
NEMI: National environmental approaches index
AGP: Assessment of green profile
GAPI: Green analytical procedure index
PLS: Partial least squares regression
PCR: Principal component regression
ANN: Artificial neural network
MCR-ALS: Multivariate curve resolution-alternating least squares
HPLC: High performance liquid chromatography
LVs: Latent variables
RMSEC: Root mean square calibration error
FWHM: Full width at half maximum
MSE: Mean squared error
RMSEP: Root mean square error of prediction
μg: Microgram
mL: Milliliter
nm: Nanometer
ICH guidelines: International council on harmonization
SD: Standard deviation
RSD: Relative standard deviation
